# *BMC Psychology* reviewer acknowledgement 2015

**DOI:** 10.1186/s40359-016-0112-9

**Published:** 2016-02-10

**Authors:** Anna Clark

**Affiliations:** BioMed Central, Floor 6, 236 Gray’s Inn Road, London, WC1X 8HB UK

## Abstract

The editors of *BMC Psychology* would like to thank all our reviewers who have contributed to the journal in Volume 3 (2015).

**Richard Adams**

The Netherlands

**Eileen Anderson-Fye**

USA

**Lorenzo Avanzi**

Italy

**Daniel Baker**

UK

**Courtney Baker**

USA

**Baptiste Barbot**

USA

**Hendrik Berth**

Germany

**Ilse Blignault**

Australia

**India Bohanna**

Australia

**Chiara Buizza**

Italy

**Brendan Bunting**

UK

**Ana Butković**

Croatia

**Emanuele Antonio Maria Cappella**

Italy

**Joseph Chilcot**

UK

**Mark Corrigan**

Ireland

**Georgina Cox**

Australia

**Kim De Jong**

The Netherlands

**Matty De Wit**

The Netherlands

**Dan Denis**

UK

**Theresa Dicke**

Germany

**Samantha Dockray**

Ireland

**Hongfei Du**

China

**Stefan Duschek**

Austria

**Andreas Eickhorst**

Germany

**William Ellison**

USA

**Cynthia Fair**

USA

**Katherine Fiori**

USA

**Signe Flottorp**

Norway

**Ted C. T. Fong**

Hong Kong

**David Frank**

USA

**Fabian Gander**

Switzerland

**David Garcia-Burgos**

Switzerland

**Abigail Gewirtz**

USA

**Emma Godfrey**

UK

**Francesca Graziano**

Italy

**Carla Groh**

USA

**Daniel Gruehn**

USA

**Aysegul Selcen Guler**

Turkey

**Kurt Hahlweg**

Germany

**Åse Marie Hansen**

Denmark

**Phillipa Hay**

Australia

**Ashleigh Haynes**

Australia

**Sascha Hein**

USA

**Randall Holcombe**

USA

**Antje Horsch**

Switzerland

**Suzanne Huot**

Canada

**Sigurd William Hystad**

Norway

**Tomasz Jankowski**

Poland

**Aaron Jarden**

New Zealand

**Sameer Jauhar**

UK

**Josef Jenewein**

Switzerland

**Peter Johnston**

UK

**Sean Kang**

USA

**Natasha Khamisa**

South Africa

**Peter Kitchen**

Canada

**Dieter Kleinböhl**

Germany

**Heidi Koschwanez**

New Zealand

**Lenka Kostovicová**

Slovakia

**Emily Kothe**

Australia

**Kasia Kozlowska**

Australia

**Assaf Kron**

Israel

**Martin Lages**

UK

**Daniel Lakens**

The Netherlands

**Brian Lakey**

USA

**Marco Lauriola**

Italy

**Katharina Ledermann**

Switzerland

**Sakari Lemola**

Switzerland

**Gary Lewis**

UK

**Herman Hm Lo**

Hong Kong

**Elma Lorenzo-Blanco**

USA

**Vivian W. Q. Lou**

Hong Kong

**Sanja Lujic**

Australia

**Marta Marques**

UK

**Chantal Martin-Soelch**

Switzerland

**Barbara Maughan**

UK

**Peter Mckenna**

Spain

**Nadine Messerli**

Switzerland

**Lynn Miller**

Canada

**Mariella Miraglia**

Canada

**Paul Mkandawire**

Canada

**Martha Mohler**

USA

**Christine Moutier**

USA

**Kelly Lynn Mulvey**

USA

**Aja Murray**

UK

**Urs Nater**

Germany

**Nathalie Noret**

UK

**Sam Norton**

UK

**Fridtjof Nussbeck**

Germany

**Misari Oe**

Japan

**Claire O'Reilly**

Australia

**Nantje Otterpohl**

Germany

**Francesco Pagnini**

Italy

**Massimiliano Pastore**

Italy

**Nicola Petrocchi**

Italy

**Ron Prinz**

USA

**Diana Rancourt**

USA

**Tyler Renshaw**

USA

**Ian Robertson**

UK

**Anne Elizabeth Rogers**

UK

**Lucia Romo**

France

**Barry Rosenfeld**

USA

**Nina Rottmann**

Denmark

**Richard Rowe**

UK

**Kai Ruggeri**

UK

**Ulrich Schnyder**

Switzerland

**Enrico Schulz**

UK

**Margaret Signorella**

USA

**Janne Skakon**

Denmark

**Andria Spyridou**

Germany

**Michael Stern**

USA

**Sarah Stewart-Brown**

UK

**Lisanne Stone**

The Netherlands

**Jade Thai**

UK

**Neil Thomas**

Australia

**Penny Trayner**

UK

**Linda Van Den Berg**

Germany

**Frank Van Der Horst**

The Netherlands

**Carol Van Hulle**

USA

**Michele Vecchione**

Italy

**Harrie Vorst**

The Netherlands

**Lisa Wagner**

Switzerland

**Marco Weber**

Germany

**Yvonne Whelan**

UK

**Redford Williams**

USA

**Daniel Wright**

USA

**Hongwei Xu**

USA

**Kimberly Young**

USA

**Robert Zachariae**

Denmark

**Coralie Zumwald**

Switzerland

**Laura Zwaan**

The Netherlands

